# Toll-Like Receptor 4 Is Essential for the Expression of Sphingosine-1-Phosphate-Dependent Asthma-Like Disease in Mice

**DOI:** 10.3389/fimmu.2017.01336

**Published:** 2017-10-18

**Authors:** Fiorentina Roviezzo, Rosalinda Sorrentino, Michela Terlizzi, Maria Antonietta Riemma, Valentina Mattera Iacono, Antonietta Rossi, Giuseppe Spaziano, Aldo Pinto, Bruno D’Agostino, Giuseppe Cirino

**Affiliations:** ^1^Department of Pharmacy, School of Medicine, University of Naples Federico II, Naples, Italy; ^2^Department of Pharmacy (DIFARMA), University of Salerno, Salerno, Italy; ^3^Department of Experimental Medicine, Section of Pharmacology, University of Campania “Luigi Vanvitelli”, Naples, Italy

**Keywords:** sphingosine-1-phosphate, TLR4, lung function, Th2 environment, Allergic diseases

## Abstract

Sphingosine-1-phosphate (S1P) levels significantly increase in bronchoalveolar lavage (BAL) of asthmatic patients following segmental allergen challenge and this increase well correlates with pulmonary inflammation. Epidemiological, genetic, clinical, and experimental data indicate a potential for the toll-like receptor 4 (TLR4) to initiate and exacerbate allergic airway diseases. The aim of this study was to evaluate the contribution of TLR4 in S1P-dependent asthma-like disease in mice. BALB/c or TLR4 defective (C3H/HeJ) mice received S1P (10 ng/mouse), LPS (0.1 μg/mouse) or S1P + LPS. Furthermore, S1P-treated BALB/c mice were injected with the purified rabbit anti-TLR4 antibody (10 μg/mouse). S1P administration induced airway hyperreactivity and pulmonary inflammation associated to an increase in the percentage of dendritic cells (DCs) and macrophages into the lung of BALB/c mice. These effects were coupled to a reduction of DCs in the mediastinic lymph node. All these S1P-mediated effects were absent in TLR4 defective mice or reversed by treatment with a purified rabbit anti-TLR4 antibody. Confocal analysis of pulmonary sections showed a significant increase in TLR4^+^ cells and a similar presence of S1P_1_ and TLR4 following S1P challenge. Accordingly, the immunoprecipitation evidenced an increased S1P_1_/TLR4 interaction. In conclusion, our findings suggest that a functional interaction between S1P_1_ and TLR4 leads to an enhanced allergic inflammatory response. Thus, S1P pathway contributes to the sentinel role played by innate immunity providing new targets for prevention and treatment of allergic airway diseases.

## Introduction

Sphingosine-1-phosphate (S1P) is generated from the cell membrane component sphingomyelin (1). S1P levels are regulated by the balance between its synthesis through sphingosine kinases and its degradation by the S1P lyasi (2–4). S1P binds plasma membrane G-protein-coupled receptors or acts on intracellular targets (5, 6). It has been widely demonstrated that the inhibition of S1P biosynthesis reduces allergen-induced asthma-like features in the mouse (7). Conversely, S1P administration exacerbates antigen-induced airway inflammation and hyperresponsiveness (8). In perfect tune, clinical studies show that S1P levels significantly increase in bronchoalveolar lavage (BAL) of asthmatic patients following segmental allergen challenge and that this increase well correlates with pulmonary inflammation (9). In addition, genome-wide associated studies have linked Orm protein, negative regulators of sphingolipid metabolism to childhood onset asthma (10, 11). S1P signaling has long been known to mediate lymphocyte trafficking in immunity and allergy by promoting lymphocytes migration (12, 13). Recently it has been defined an intrinsic function for S1P and its receptors in both innate and adaptive immune system independently from immune cell trafficking (14). Systemic administration of S1P, without adjuvant factors, promotes in the mouse a disease closely mimicking the cardinal features of severe asthma in humans such as pulmonary eosinophil inflammation, high circulating levels of IgE, and predominance of CD4^+^ T cell-derived IL-4 (15–17). On the other hand, local application of FTY720, acting as functional antagonist of S1P_1_ reverses experimental asthma by modulating dendritic cell (DC) function (18).

In the last decades, asthma has become epidemic, associated with increases of mortality rates (19). This increase in industrialized countries suggests that environmental factors/pollution may contribute to the pathogenesis of asthma (20). For a long time, the improved hygiene standards, coupled to a less frequent microbial infections and/or exposure to microbial partial structures, have been thought to skew the immune system toward a Th2 phenotype, which is associated to the development of atopic diseases (21, 22). Conversely, the Th1 skewing cytokines released in response to invading microbes protect from asthma (20, 23). However, more several independent lines of evidence suggest that the hygiene hypothesis may over-simplify the role of pathogen-derived signals in allergic disease development and modulation (20). Indeed certain respiratory infections favor asthma development rather than protect (24). In this context, a key role is played by toll-like receptor 4 (TLR4), which function is required to develop allergic response (25–29). Many association studies have reported that TLR polymorphisms and in particular TLR4 predispose to allergic diseases (30, 31).

First evidence that TLR4 may promote airway sensitization and eosinophilic airway inflammation comes from studies on mice that lack functional TLR4, C3H/HeJ (27, 32, 33). These mice failed to develop the main features of asthma following sensitization because of an impaired immune response. These studies demonstrated that TLR4 function is likely to be required also in the airway epithelia, which might undermine the barrier function of the epithelium (27, 34). Nevertheless, asthmatic subjects with high total serum IgE show increased macrophage expression of TLR4 in induced sputum (35). Thus, the aim of this study was to investigate the role of TLR4 in asthma-like features such as inflammatory infiltrate, airway hyperreactivity, and mucus exacerbation in mice exposed to S1P.

## Materials and Methods

### Animals

Female BALB/c and C3H/HeJ (TLR4 defective) mice were purchased by Charles River (Italy). The animals were housed in a controlled environment and provided with standard rodent chow and water. All animals were allowed to acclimate for 4 days prior to experiments and were subjected to 12 h light–12 h dark schedule. Experiments were conducted during the light phase. The experimental procedures, according to Italian (DL 26/2014) and European regulations (no. 63/2010/UE) on the protection of animals used for experimental and other scientific purposes, were approved by the Italian Ministry.

### S1P Treatment Protocol

BALB/c and C3H/HeJ (TLR4 defective) mice received at days 0 and 7 subcutaneous (s.c.) injection of S1P (10 ng; Enzo Life Science, Italy) dissolved in sterile saline containing bovine serum albumin (0.001%) according to the manufacturer’s instructions. Subcutaneous administration of S1P induces in BALB/c mice asthma-like disease (17). As previously shown, these effects are sustained by a Th2 response as confirmed by the fact that the adoptive transfer of CD4^+^ T cells obtained from mice injected with S1P increases bronchial reactivity and induces pulmonary inflammation of recipient (non-treated) mice (17). In another set of experiments, BALB/c or C3H/HeJ (TLR4 defective) mice received lipopolysaccharide (0.1 µg; intranasal instillation LPS from *Escherichia coli* 0111:B4; Sigma Aldrich, Italy) or S1P (10 ng) or the association LPS + S1P at day 0 and 7. Another group of S1P-treated mice received the purified rabbit anti-TLR4 (10 µg; i.p. H-80, sc-10741; Santa Cruz) 30 min prior to S1P administration. Mice were sacrificed at days 10 and 21 and bronchi and lungs harvested and used to perform functional and molecular studies.

### Measurement of Airway Hyperreactivity

Bronchial rings of 1–2 mm length were cut and placed in organ baths connected to isometric force transducers (Type 7006, Ugo Basile, Comerio, Italy) and to a Powerlab 800 (AD Instruments, Ugo Basile, Comerio, Italy). Briefly, rings were initially stretched until a resting tension of 0.5 g was reached and allowed to equilibrate for at least 30 min. Bronchial rings were challenged with carbachol (10^−6^ mol/L) until the response was reproducible. Once a reproducible response was achieved, bronchial reactivity was assessed performing a cumulative concentration–response curve to carbachol (1 × 10^−8^–3 × 10^−5^ mol/L).

### Isolated Perfused Mouse Lung Preparation

Lung function was assessed using an isolated and perfused mouse lung model (36). Lungs were perfused in a non-recirculating fashion through the pulmonary artery at a constant flow of 1 ml/min resulting in a pulmonary artery pressure of 2–3 cm H_2_O. The perfusion medium used was RPMI 1640 lacking phenol red (37°C). The lungs were ventilated by negative pressure (−3 and −9 cm H_2_O) with 90 breathmin^−1^ and a tidal volume of about 200 µl. Every 5 min, a hyperinflation (−20 cm H_2_O) was performed. Artificial thorax chamber pressure was measured with a differential pressure transducer (Validyne DP 45-24) and airflow velocity with pneumotacho graph tube connected to a differential pressure transducer (Validyne DP 45-15). The lungs respired humidified air. The arterial pressure was continuously monitored by means of pressure transducer (Isotec Health dyne), which was connected with the cannula ending in the pulmonary artery. All data were transmitted to a computer and analyzed with Pulmodyn software (Hugo Sachs Elektronik, March Hugstetten, Germany). Data were analyzed through the following formula: *P* = *V*·*C*^−1^ + *R*_L_·*dV*·*dt*^−1^, where *P* is chamber pressure, *C* pulmonary compliance, *V* tidal volume, *R*_L_ airway resistance. Successively, airway resistance value registered was corrected for the resistance of the pneumotacometer and the tracheal cannula of 0.6 cm H_2_O s ml^−1^. Lungs were perfused and ventilated for 45 min without any treatment to obtain baseline state. Subsequently, lungs were challenged with carbachol. Repetitive dose response curve of carbachol was administered as 50 µl bolus, followed by intervals of 15 min in which lungs were perfused with buffer only.

### Western Blotting Analysis

Lungs were homogenized in ice-cold lysis buffer containing a protease inhibitors and 0.1% Triton X-100. Samples (40 μg/lane) were separated on a 10% SDS-PAGE gel and transferred to a PVDF membrane. Membrane were blocked by incubation in PBS containing 0.1% v/v Tween 20 and 5% non-fat dry milk for 2 h, followed by a overnight incubation at 4°C with rabbit polyclonal anti-TLR4 (1:1,000; H-80, sc-10741; Santa Cruz) or rabbit monoclonal anti-S1P_1_ (1:1,000, ab125074 Abcam, UK) The filters were washed with PBS containing 0.1% v/v Tween 20 and incubated for 2 h with anti-horseradish peroxidase-conjugate secondary antibody. Membranes were washed and developed using enhanced chemiluminescence substrate (ECL). The band intensity was quantified by densitometric analysis using Image J analysis program and normalized to GADPH expression.

### Immunoprecipitation (IP)

Lungs were omogenized and the cell lysate was pre-cleared by adding 100 µl of protein A/G plus-Agarose (Santa Cruz) for 1 ml of cell lysate. The protein A/G beads were removed by centrifugation. The immunoprecipitating antibody (20 µg monoclonal rabbit anti-S1P_1_; Abcam, UK) was added to 500 µl of cell lysate. The cell lysate/antibody mixture was gently rocked overnight at 4°C. The immunocomplex was extracted by adding 100 µl protein A agarose/sepharose bead slurry and gently rocking on either a orbital shaker for 1 h at 4°C. The agarose/sepharose beads were collected by centrifugation. The supernatant was recovered [eluted proteins (EP)] and the beads washed three times with 800 µl ice-cold modified RIPA buffer. The protein complexes immunoprecipitation (IP) and the proteins eluted (EP) from beads incubated with lysate and S1P_1_ antibody were suspended in the sample buffer and boiled for 5 min. The denatured proteins were separated on 10% SDS polyacrylamide gel and transferred to a PVDF membrane. Membrane were blocked by incubation in PBS containing 0.1% v/v Tween 20 and 5% non-fat dry milk for 2 h, followed by a overnight incubation at 4°C with anti-TLR4, before incubation for 2 h with anti-horseradish peroxidase-conjugate secondary antibody. Membranes were then developed using ECL. The band intensity was quantified by densitometry using Image J analysis program and normalized to IgG for IP and to GADPH for EP.

### Immunofluorescence Analyses

Left lung lobes were paraffin-embedded. Cryosections were stained for TLR4-AlexaFluor 488 (Invitrogen, CA, USA) or S1P1-AlexaFluor 555 (Invitrogen, CA, USA). Nuclei were counter-stained with DAPI (Sigma Aldrich, Rome, Italy). Images were observed by means of Carl Zeiss confocal microscopy (magnification: 40×).

### Flow Cytometry Analysis

Lungs were isolated and digested with 1 U/ml collagenase (Sigma Aldrich, Milan, Italy). Cell suspensions were passed through 70 µm cell strainers, and red blood cells were lysed. Cell suspensions were used for flow cytometric analysis of different cell subtypes (37, 38). The composition of lung inflammatory cells was determined by flow cytometry (BD FacsCalibur Milan, Italy) using the following antibodies: CD11c-APC, CD11b-PeCy5.5, F4/80-PE, MHC-FITC, MHC II-PE (Bioscience, San Diego, CA, USA). Appropriate isotype controls were used.

### Immunohistochemistry

Left lung lobes were fixed in OCT medium (Pella Inc., Milan, and Italy) and 7 µm cryosections were cut. The degree of inflammation was scored by blinded observers by using Periodic acid/Alcian blue/Schiff staining (PAS). PAS staining (Sigma Aldrich, Milan Italy) was performed according to the manufacturer’s instructions to detect glycoprotein. PAS^+^ cryosections were graded with scores 0 to 4 to describe low to severe lung inflammation as follows: 0: <5%; 1: 5–25%; 2: 25–50%; 3: 50–75%; 4: <75% positive staining/total lung area. In another set of experiments, anti-TLR4 antibody (Santa Cruz, CA, USA) was used to perform immunohistochemistry analysis. The diammino-benzidinic acid (DAB) system was used to detect complexes. Rat IgG was used as an isotype control (Santa Cruz, CA, USA). TLR4 positive staining was expressed as media of μm^2^/field (at least five field were analyzed for each sample).

### Statistical Analysis

The statistical analysis was performed using the prism package Graph-Pad software (version 5, San Diego, CA, USA). Data were expressed as mean ± SEM from six mice in each group. The level of statistical significance was determined by one-way or two-way ANOVA followed by the Bonferroni test.

## Results

### S1P Fails to Induce Airway Hyperreactivity in C3H/HeJ Mice (TLR4 Defective)

**Figure 1:**
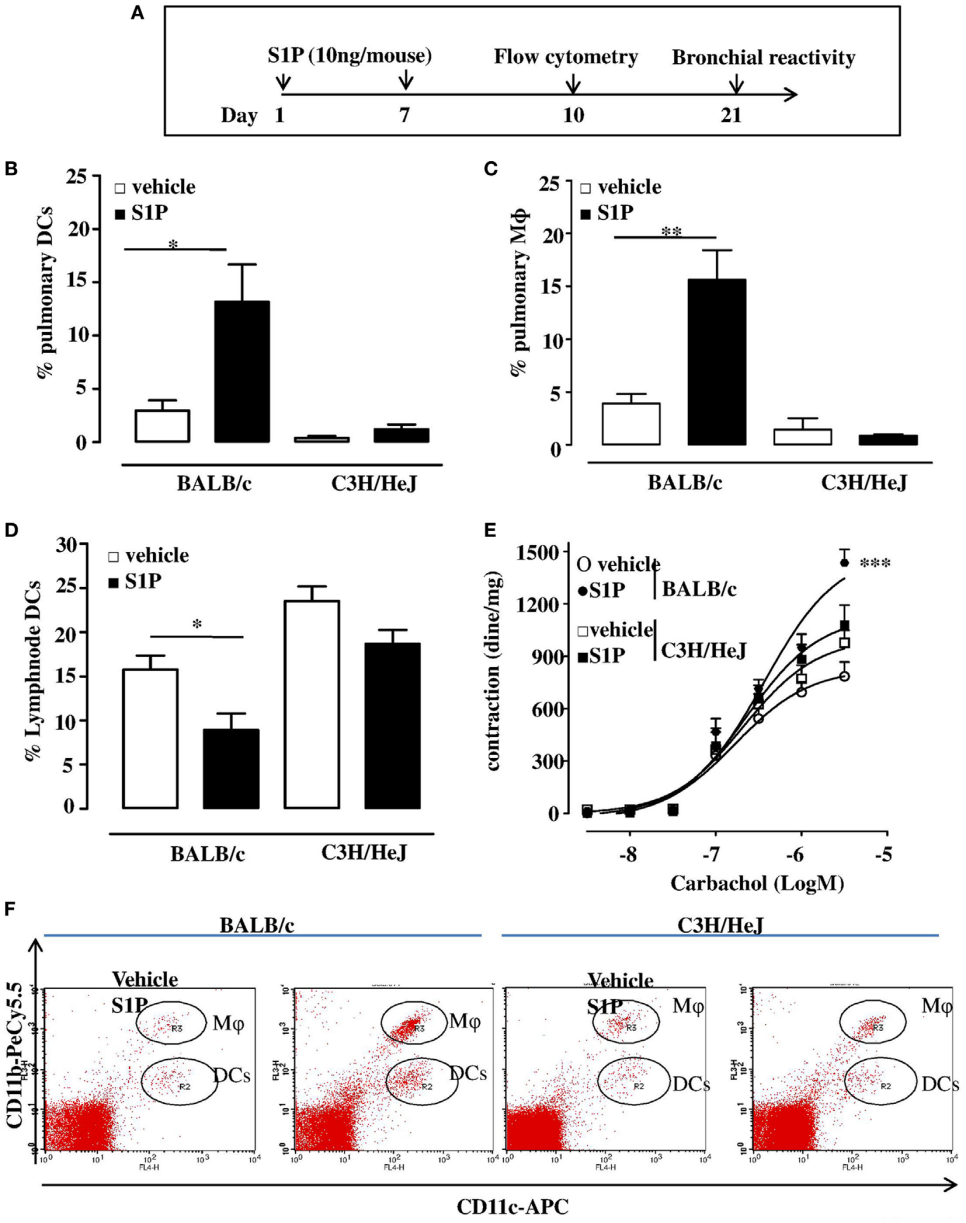
**(A)** Treatment protocol: BALB/c or C3H/HeJ (toll-like receptor 4 defective) mice received sphingosine-1-phosphate (S1P) 10 ng/sc per mouse at days 0 and 7 or vehicle. At 10 days, pulmonary dendritic cell (DC), e.g., CD11c^+^CD11b^int^ F4/80^−^ cells **(B)** and macrophage (Mφ,), e.g., CD11c^+^CD11b^high^ F4/80^+^ cells **(C)** recruitment was evaluated into the lung of S1P-treated BALB/c mice and C3H/HeJ mice. **(D)** Percentage of DCs in lymph node harvested from S1P-treated BALB/c or C3H/HeJ mice. **(E)** Bronchial reactivity to carbachol at 21 days. **(F)** Representative flow cytometry dot plots. All data are expressed as mean ± SEM from *n* = 6 animals for each group. Data were analyzed using Student’s *t*-test **(B,C,D)** or two-way ANOVA followed by Bonferroni **(E)**; *p < 0.05; **p < 0.01; ***p < 0.001 vs each corresponding vehicle.

Subcutaneous administration of S1P (10 ng/mouse; Figure 1A) induced an increase in the percentage of DCs within the lung that were identified as CD11c^+^CD11b^int^ F4/80^−^ cells (Figure 1B) as well as a significant increase in macrophage (Mφ), identified as CD11c^+^CD11b^high^ F4/80^+^ cells infiltration (Figure 1C). These effects were absent when S1P was administered to C3H/HeJ mice (Figures 1B,C). In addition, we observed a significant reduction of percentage of DCs within mediastinic lymph node (LNs) in S1P-challenged BALB/c mice (Figure 1D) as opposite to C3H/HeJ mice (Figure 1D). A representative flow cytometry dot plot is reported in Figure 1F. In perfect tune with these data, S1P induced airway hyperreactivity only in BALB/c mice (Figure 1E), while it did not affect the bronchial tone in C3H/HeJ mice (Figure 1E).

### LPS Potentiates S1P-Induced Bronchial Hyperreactivity and Exacerbates Lung Damage

**Figure 2:**
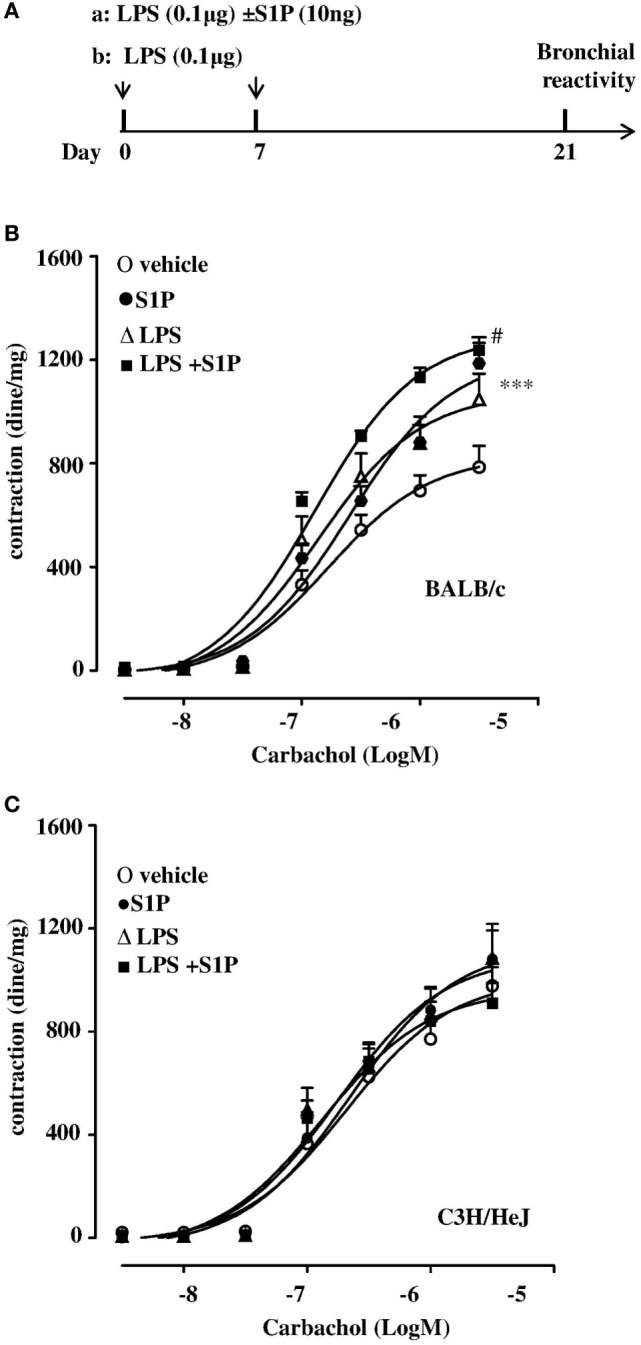
**(A)** Treatment protocol:BALB/c and C3H/HeJ (toll-like receptor 4 defective) mice received LPS (0.1 μg/mouse, protocol a) alone or together with sphingosine-1-phosphate (S1P) (10 ng, protocol b) at days 0 and 7, or S1P as described in Figure 1A. Bronchial reactivity to carbachol at day 21 was evaluated in BALB/c **(B)** or C3H/HeJ **(C)**. Data are expressed as mean ± SEM from *n* = 6 animals for each group. Data were analyzed using two-way ANOVA followed by Bonferroni; ^#^*p* < 0.05vs S1P; ***p < 0.001 vs vehicle.

LPS, administered at the dose of 0.1 μg/mouse at day 0 and day 8 (Figure 2A), induced a significant increase in carbachol-mediated bronchial contraction, when compared to vehicle (Figure 2B). In addition, the co-administration of S1P (10 ng/mouse) and LPS (0.1 μg/mouse) induced a further significant increase in bronchial reactivity when compared to mice treated separately with S1P or LPS (Figure 2B). LPS or S1P alone or the combination S1P + LPS did not induce any effect on bronchi harvested from C3H/HeJ mice (Figure 2C).

**Figure 3:**
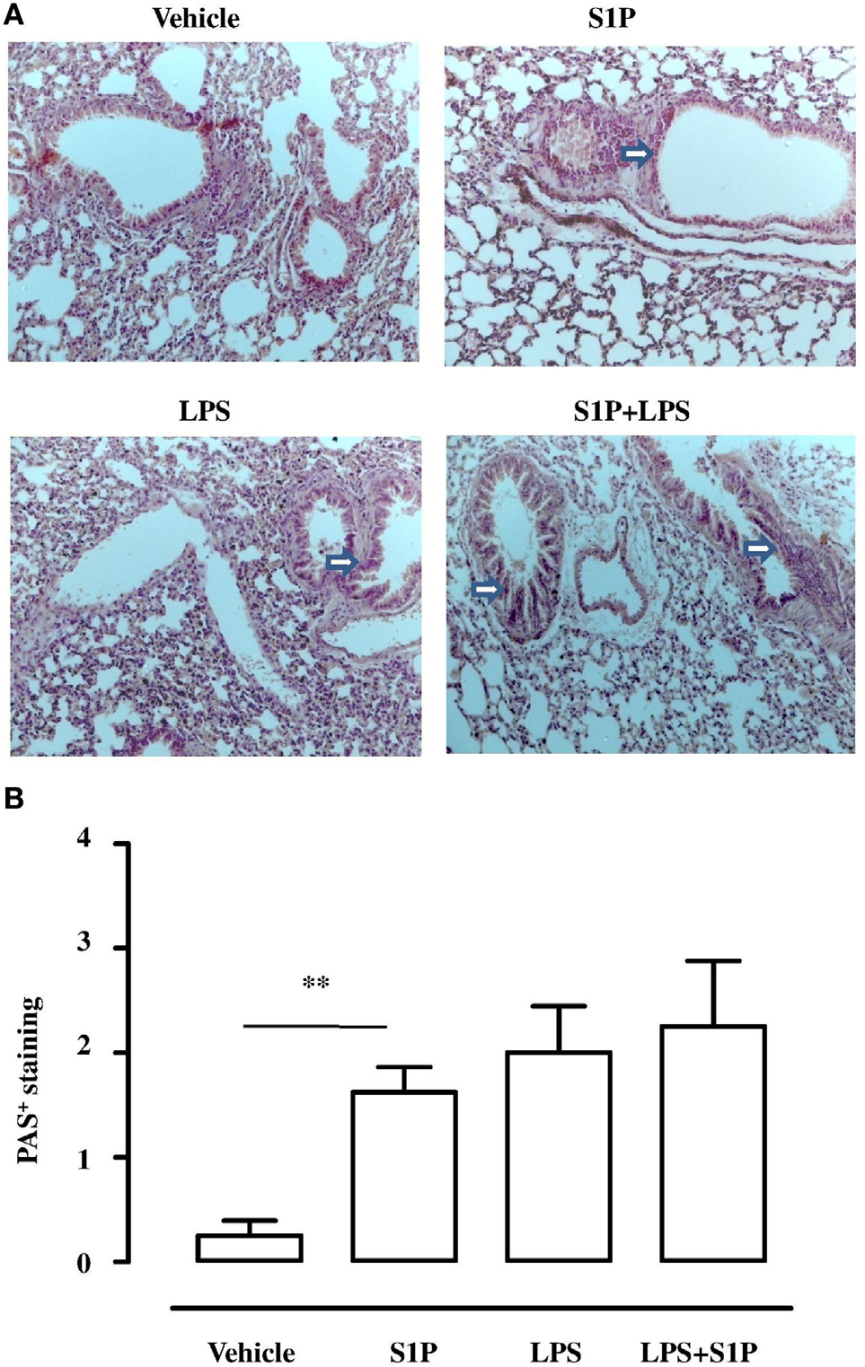
**(A)** Representative Periodic acid/Alcian blue/Schiff staining (PAS) performed on lung cryosection from BALB/c mice treated with vehicle, sphingosine-1-phosphate (S1P) (10 ng/mouse), LPS (0.1 μg/mouse) and S1P + LPS. **(B)** Quantitative analysis of immunohistochemistry of PAS^+^ staining (magnification 40×). PAS^+^ cryosections were graded with scores 0–4 to describe low to severe lung inflammation as follows: 0: <5%; 1: 5–25%; 2: 25–50%; 3: 50–75%; 4: <75% positive staining/total lung area. Data were analyzed using Student’s *t*-test; **p < 0.01 vs vehicle.

Lungs, harvested from BALB/c mice receiving S1P together with LPS, displayed an altered bronchial structure, which showed (white arrows, Figure 3A) higher thickness than vehicle, and mice treated with the sole S1P or LPS. This marked hyperplasia was associated with higher mucus production as determined by PAS staining quantification (Figure 3B).

### S1P Induces TLR4 Upregulation in the Lung

**Figure 4:**
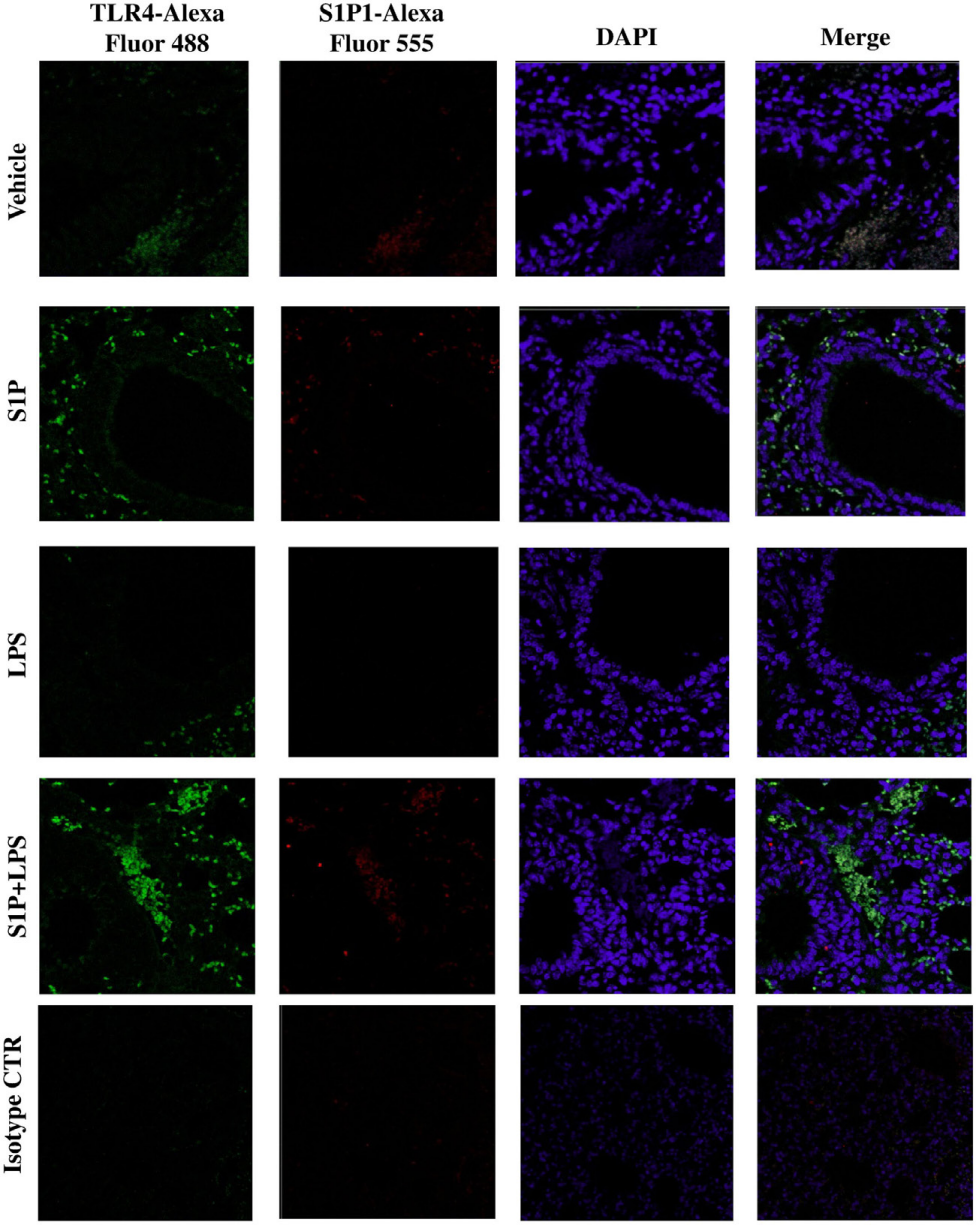
Confocal representative images for toll-like receptor 4-AlexaFluor 488, S1P1-AlexaFluor 555, and DAPI on lung section from BALB/c mice treated with vehicle, sphingosine-1-phosphate (S1P) (10 ng/mouse), LPS (0.1 μg/mouse) and S1P + LPS (magnification: 40×).

Confocal analysis of S1P_1_ and TLR4 was performed on pulmonary sections obtained by mice treated as described above (Figure 2A). A significant increase in TLR4^+^ cells occurred in the lung of S1P-treated mice with respect to vehicle (Figure 4). Interestingly, the number of TLR4^+^ cells increased when mice received the association LPS + S1P as compared to LPS or S1P alone (Figure 4). No difference in S1P_1_ expression was evidenced in the lungs of vehicle, LPS alone, S1P alone compared to LPS together with S1P, which showed higher levels of S1P1^+^ cells (Figure 4).

### S1P Promotes TLR4/S1P_1_ Association

**Figure 5:**
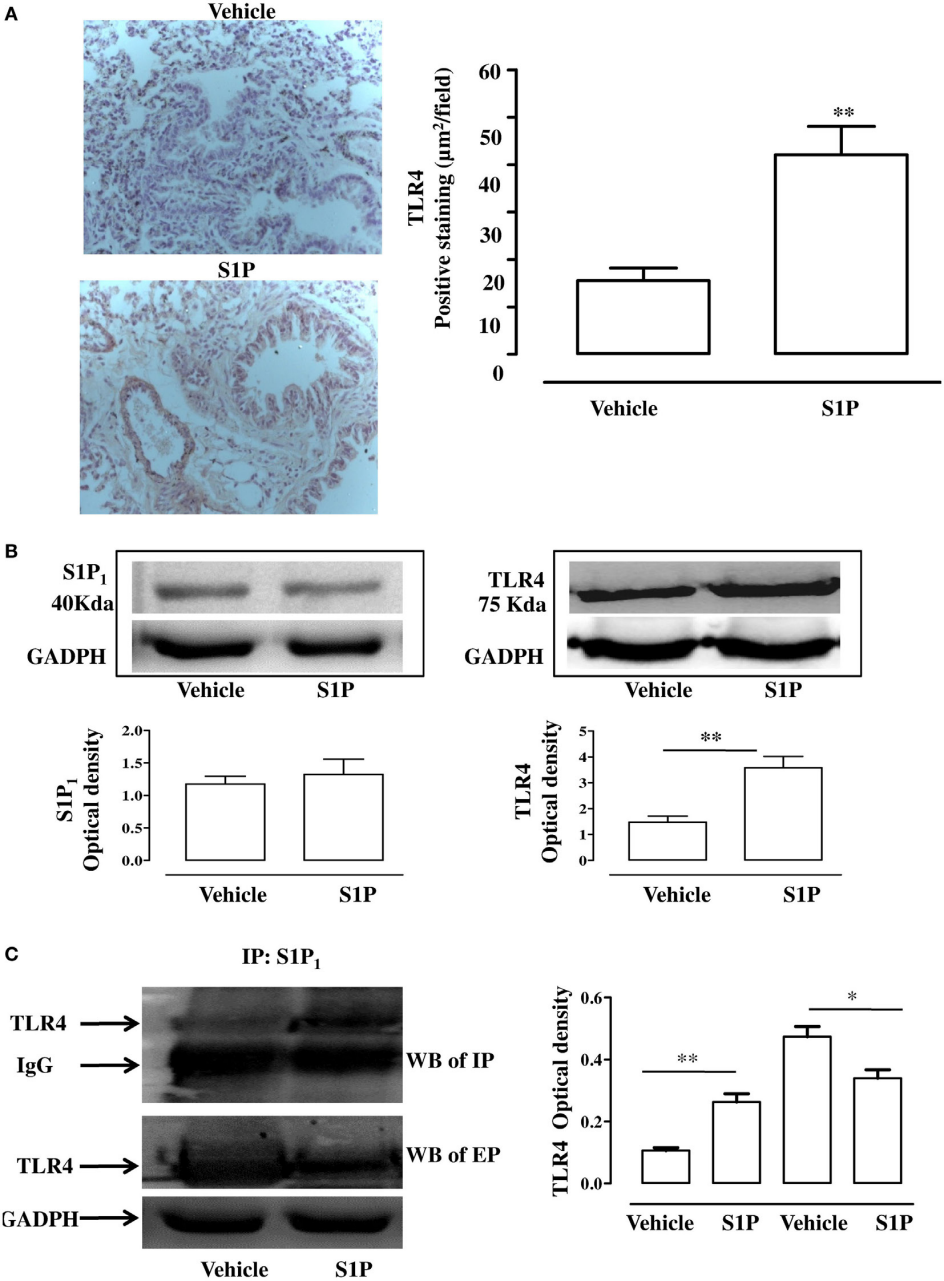
**(A)** Immunohistochemical analysis: representative images (left panel) and quantitative analysis for toll-like receptor 4 (TLR4) detection (right panel) in lung cryosection of sphingosine-1-phosphate (S1P)- or vehicle-treated BALB/c mice (Magnification: 40×). **(B)** Expression of S1P_1_ (left panel) and TLR4 (right panel) evaluated by western blot in total cell lung lysates. Densitometric analysis has been normalized to GADPH expression. **(C)** Western blot of TLR4 (left panel) in cell lysates of lung immunoprecipitation (IP) with S1P_1_ and in the eluted proteins (EP) from beads incubated with lysate and with S1P_1_ antibody. Densitometric analysis (left panel) has been normalized to IgG or GADPH expression in IP and western blot, respectively. Data were analyzed using Student’s *t*-test; **p < 0.01; *p < 0.05.

Immunohistochemistry analysis performed on pulmonary sections harvested from S1P-treated mice confirmed a significant increase in TLR4^+^ staining (Figure 5A). TLR4 expression was more intense in proximity of the bronchi harvested from S1P-treated mice as compared to vehicle (Figure 5A). In perfect tune with these data, western blot confirmed an increased expression of TLR4 in bronchi harvested from S1P-treated mice compared to vehicle (Figure 5B), while S1P_1_ expression was not affected (Figure 5B). Immunoprecipitation performed with the antibody for S1P_1_ showed an increased interaction with TLR4 in the lung following exposure of mice to S1P (Figure 5C). In support, the western blot performed on the eluted fraction (EP) of immunoprecipitation demonstrated an increased expression of TLR4 in vehicle as compared to S1P stimulated conditions (Figure 5C).

### TLR4 Neutralizing Antibody Inhibits S1P-Induced Airway Hyperreactivity and Pulmonary Inflammation

**Figure 6:**
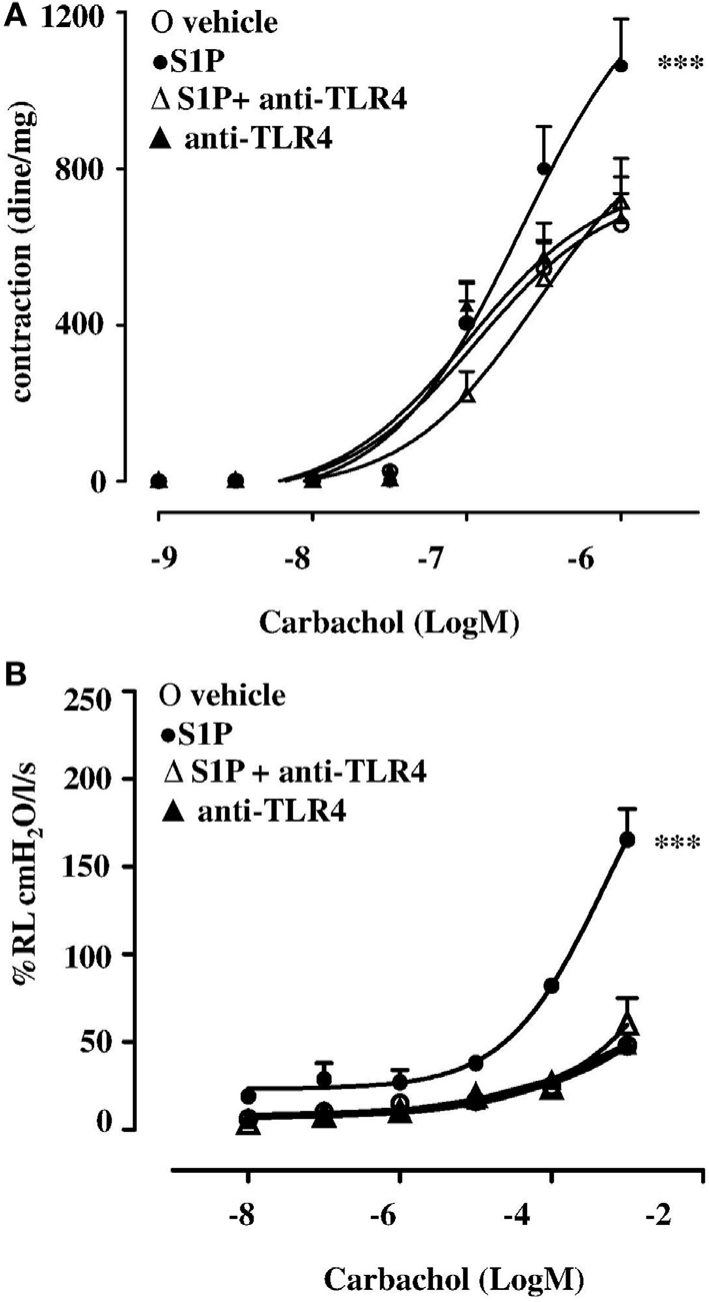
BALB/c mice received 30 min prior to sphingosine-1-phosphate (S1P) or vehicle intraperitoneal administration of the purified rabbit anti-TLR4 (10 μg) at days 0 and 7 (*n* = 6). Bronchial reactivity **(A)** and lung resistance **(B)** were evaluated at 21 days. Data are expressed as mean ± SEM from *n* = 6 animals for each group. Data were analyzed using two-way ANOVA followed by Bonferroni; ***p < 0.001 vs vehicle.

**Figure 7:**
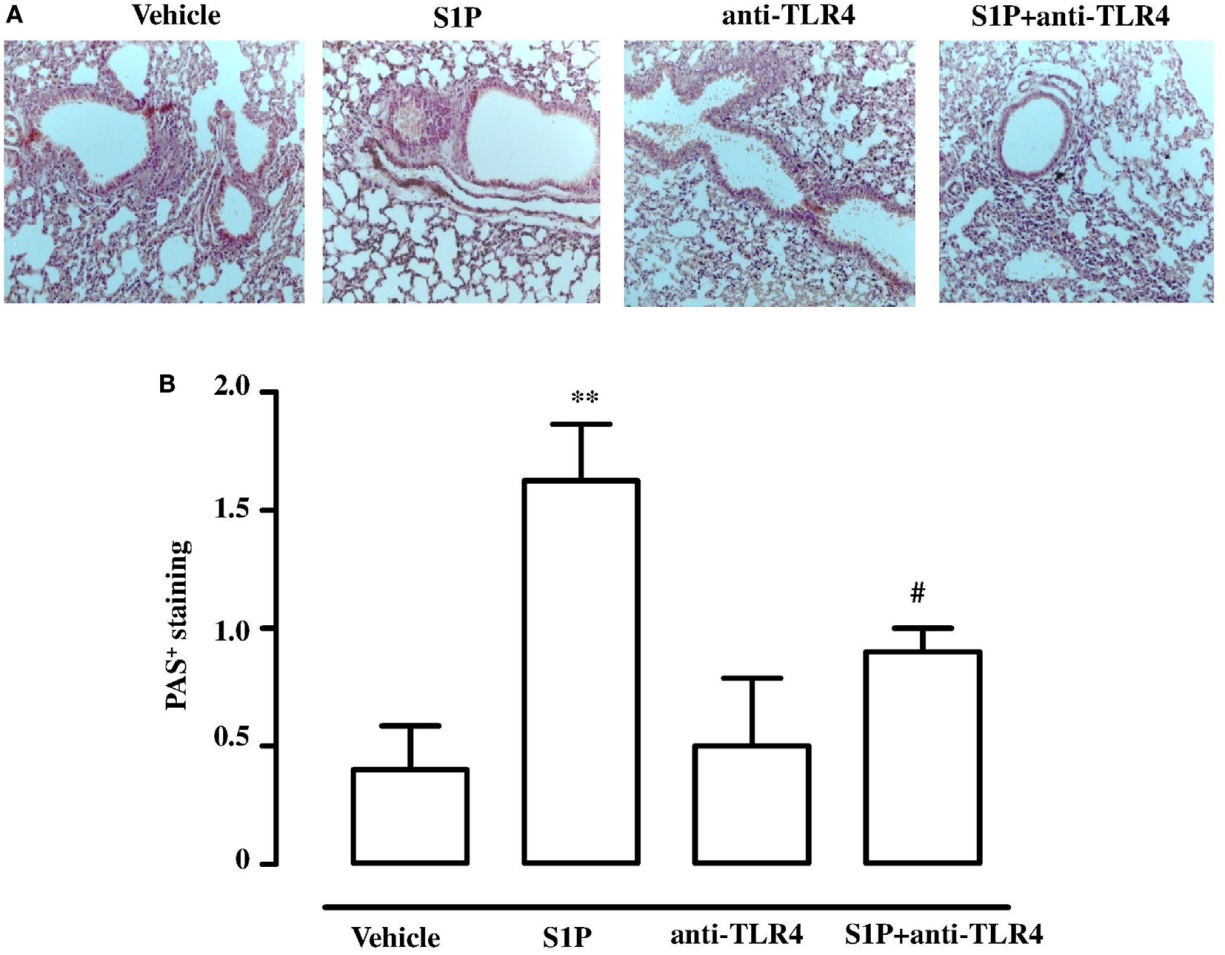
**(A)** Representative Periodic acid/Alcian blue/Schiff staining (PAS) performed on lung cryosection from BALB/c mice treated with vehicle, sphingosine-1-phosphate (S1P) (10 ng/mouse), antibody against TLR4 (anti-TLR4), and S1P + anti-TLR4 (10 µg) 30 min prior to S1P. **(B)** Quantitative analysis of immunohistochemistry of PAS^+^ staining (Magnification: 40×). Data were analyzed using Student’s *t*-test; **p < 0.01 vs vehicle; ^#^*p* < 0.05 vs S1P.

In order to further asses the role of TLR4, we pre-treated BALB/c mice with a TLR4 neutralizing antibody. TLR4 neutralizing antibody abrogated S1P-induced bronchial hyperreactivity (Figure 6A). To evaluate whether this effect was related to the whole lung function, pulmonary resistances (R_L_) was measured in anesthetized, tracheotomized, and ventilated mice using a whole-body plethysmography. R_L_ were significantly increased in the response to carbachol in S1P-mice that was abrogated by the TLR4 neutralizing antibody (Figure 6B). In support, PAS staining performed on lung sections evidenced a significant increase in mucus production following S1P treatment that significantly inhibited the TLR4 neutralizing antibody (Figures 7A,B).

## Discussion

The exact mechanism by which major allergens are recognized by the host have not been completely clarified (39), but recently it has been demonstrated that TLR4 plays a key role in the response to common allergens, i.e., house dust mite protein (26) and the metal nickel (28). Moreover, it has been also hypothesized that TLR4 may be activated by endogenous molecules released by damaged cells or chemical substances generated during tissue injury in severe inflammatory processes (40). Preclinical and clinical studies show a clear involvement of S1P in the pathogenesis of chronic asthma (41). S1P levels increase in lung tissues after allergen challenge in experimental models of asthma as well as in the BAL fluid of asthmatic patients. In addition, systemic administration of S1P by itself, without additional adjuvant factors, triggers in the mouse a disease closely mimicking the main features of severe asthma in humans, such as bronchial hyperreactivity (7, 15, 17, 42).

We have previously shown that the systemic administration of S1P to BALB/c mice triggers an increase in airway reactivity and pulmonary inflammation that is dependent upon a Th2 response as confirmed by a specific adoptive transfer experiment using CD4^+^ T cells harvested from S1P-treated BALB/c mice (17). In order to address the involvement of TLR4 in S1P-induced response in the lung, we administered S1P to C3H/HeJ (TLR4 defective mice). In TLR4 defective mice, S1P resulted inefficacious. Indeed, S1P failed to induce DC and macrophage lung infiltration as well as to modify the percentage of DCs into the mediastinic LN. Therefore, it is feasible that TLR4 plays a key role in S1P effects on the immune pulmonary environment and participates in the molecular framework influencing the airway function. This hypothesis well fits with the finding that in the functional studies, performed on airways of TLR4 defective mice, no changes in both bronchial reactivity and lung resistance occurred following S1P administration. TLR4 defective mice are not prone to allergic pulmonary diseases. Indeed, TLR4 acts on both innate immune cells and the airway epithelia, which need to communicate each other to cause allergic disease. Although it has been shown that TLR4 contributes to Th_2_ responses, Millien et al. (43) have demonstrated that TLR4 is not essential for Th2 response, but rather is necessary for innate airway cells responsiveness to Th_2_ cells. Overall, these data point toward a key role of TLR4 in S1P effects on both the immune arm and the epithelial/parenchymal function.

In allergic diseases, microbial products regulate the Th1 and Th2 responses through TLR4 (44, 45). In particular, when LPS is administered at high dose, TLR4 modulates the Th response toward Th1, conversely, when LPS dose is low, promotes a Th2 response (29). Taking advantage of this synergism, we evaluated the effect of the co-administration of LPS and S1P. Treatment of BALB/c mice with S1P together with a low dose of LPS induced a significant increase in bronchial reactivity as compared to mice treated with either S1P or LPS alone. Accordingly, immunohistochemistry showed that airway inflammation in BALB/c mice was more pronounced in mice treated with the association S1P plus LPS. Again, we did not observe any effect in TLR4 defective mice. Finally, we mimicked the effect observed in TLR4 defective mice by treating BALB/c mice with a TLR4 antagonist. As expected, TLR4 blockade suppressed S1P-induced hyperreactivity and significantly inhibited pulmonary inflammation further supporting the involvement of TLR4 in sensing S1P and to mediate the pulmonary inflammatory response.

Toll-like receptor 4 signaling is under several regulatory control mechanisms, such as cooperation with co-receptors, post translational modifications, cleavage, cellular trafficking, and interactions with negative regulators (46). Much has been learned about the complex signaling pathway downstream of TLRs in the past years. In this context, lipid rafts, sphingolipid-rich microdomains, are recognized as platforms of TLR4 activation. In particular, it has been demonstrated that LPS exposure triggers TLR4 assembly on lipid rafts and sphyngomyelinase activity is essential for this process (47). Accordingly, confocal analysis of pulmonary sections evidenced a significant increase in TLR4^+^ cells in the lung of S1P-treated mice as compared to the vehicle. In particular, TLR4 expression was significantly increased in the proximity of bronchi. These results indicate the involvement of TLR4 in triggering bronchial hyperreactivity and exacerbation of pulmonary inflammation elicited by S1P. These data well fit with previously published evidences of a functional cooperation between S1P_1_ and TLR4 to promote inflammation (48, 49).

Sphingosine-1-phosphate and S1P receptors are ubiquitously expressed. S1P–S1PR signaling has been well characterized in immune trafficking and activation in innate and adaptive immune systems. In this context, a key role has been attributed to S1P/S1P_1_ signaling as also demonstrated by the therapeutic actions of fingolimod (FTY720/Gilenya), that exerts its lymphopenic effect by acting on S1P receptors, but primarily on S1P receptor 1 (S1P_1_). According to our hypothesis, we found that TLR4^+^ and S1P_1_^+^ cells further increased within the lung environment following the co-administration of LPS plus S1P. This effect was accompanied by a similar distribution of S1P_1_ and TLR4, as shown by the confocal analysis. The immunoprecipitation confirmed the existence of an interaction between S1P_1_ and TLR4 following S1P challenge. Thus, S1P induces not only TLR4 upregulation but also promotes TLR4/S1P_1_ interaction in the lung, suggesting a role of S1P in cellular compartmentalization/activation of TLR4.

Toll-like receptor 4 is not required for T_H_2 cell development but rather is required for responsiveness of innate airway cells to T_H_2 cells. In conclusion, these findings demonstrate that S1P action relies on a cooperation between S1P_1_ and TLR4. The essential role of TLR4 is defined by the lack of S1P effects in TLR4 defective mice as well as by the neutralizing effect of TLR4 antibody in BALB/c mice. The identification of this mechanism implies a role for S1P pathway in the innate immune system that is intrinsically linked with allergy. Thus, we suggest that S1P participates in the sentinel role played by the innate immunity providing new targets for prevention and treatment of chronic airway diseases.

## Ethics Statement

The experimental procedures, according to Italian (DL 26/2014) and European regulations (no. 63/2010/UE) on the protection of animals used for experimental and other scientific purposes, were approved by the Italian Ministry.

## Author Contributions

FR, RS, and GC designed the experiments and wrote the manuscript; MT and GS performed and analyzed histology experiments; VI, MR, AR, and GS performed animal and biochemical experiments; AP and BD revised the manuscript. All authors read and approved the final manuscript.

## Conflict of Interest Statement

The authors declare that the research was conducted in the absence of any commercial or financial relationships that could be construed as a potential conflict of interest.
